# Trial outcomes and information for clinical decision-making: a comparative study of opinions of health professionals

**DOI:** 10.1186/s13063-016-1492-0

**Published:** 2016-07-25

**Authors:** Angus G. K. McNair, Sara T. Brookes, Robert N. Whistance, Rachael O. Forsythe, Rhiannon Macefield, Jonathan Rees, James Jones, George Smith, Anne M. Pullyblank, Kerry N. L. Avery, Michael G. Thomas, Paul A. Sylvester, Anne Russell, Alfred Oliver, Dion Morton, Robin Kennedy, David G. Jayne, Richard Huxtable, Rowland Hackett, Susan J. Dutton, Mark G. Coleman, Mia Card, Julia Brown, Jane M. Blazeby, On behalf of the CONSENSUS-CRC (Core Outcomes and iNformation SEts iN SUrgical Studies – ColoRectal Cancer) working group

**Affiliations:** 1 Centre for Surgical Research, School of Social and Community Medicine, University of Bristol, Canynge Hall, 39 Whatley Road, Bristol, BS8 2PS UK; 2 Division of Surgery Head and Neck, University Hospitals Bristol NHS Foundation Trust, Bristol, UK; 3 Colorectal Cancer Patient Representative, North Bristol NHS Trust, Bristol, UK; 4 Department of General Surgery, North Bristol NHS Trust, Bristol, UK; 5 Severn School of Surgery, Bristol, UK; 6 Colorectal Surgery Unit, University Hospitals Bristol NHS Foundation Trust, Bristol, UK; 7 Colorectal Consumer Liaison Group, National Cancer Research Institute, London, UK; 8 Academic Department of Surgery, University of Birmingham, Birmingham, UK; 9 Department of Surgery, St Mark’s Hospital and Academic Institute, Harrow, UK; 10 Academic Surgical Unit, St James’ University Hospital NHS Trust, Leeds, UK; 11 Centre for Ethics in Medicine, University of Bristol, Bristol, UK; 12 Colorectal Site Specific Group, Somerset, Wiltshire, Avon and Gloucestershire, South West Cancer Network, Taunton, UK; 13 Centre for Statistics in Medicine and Oxford Clinical Trials Research Unit, Nuffield Department of Orthopaedics, Rheumatology and Musculoskeletal Sciences, University of Oxford, Oxford, UK; 14 Department of Colorectal Surgery, Plymouth Hospitals NHS Trust, Plymouth, UK; 15 Clinical Trials Research Unit, University of Leeds, Leeds, UK

## Abstract

**Background:**

Trials are robust sources of data for clinical practice; however, trial outcomes may not reflect what is important to communicate for decision-making. The study compared clinicians’ views of outcomes to include in a core outcome set for colorectal cancer (CRC) surgery, with what clinicians considered important information for clinical practice (core information).

**Methods:**

Potential outcome/information domains were identified through systematic literature reviews, reviews of hospital information leaflets and interviews with patients. These were organized into six categories and used to design a questionnaire survey that asked surgeons and nurses from a sample of CRC centers to rate the importance of each domain as an outcome or as information on a nine-point Likert scale. Respondents were re-surveyed (round 2) following group feedback (Delphi methods). Comparisons were made by calculating the difference in mean scores between the outcomes and information domains, and paired *t* tests were used to explore the difference between mean scores of the six outcome/information categories.

**Results:**

Data sources identified 1216 outcome/information items for CRC surgery that informed a 94-item questionnaire. First-round questionnaires were returned from 63/81 (78 %) of centers. Clinicians rated 76/94 (84 %) domains of higher importance to measure in trials than information to communicate to patients in round 1. This was reduced to 24/47 (51 %) in round 2. The greatest difference was evident in domains regarding survival, which was rated much more highly as a trial outcome than an important piece of information for decision-making (difference in mean 2.3, 95 % CI 1.9 to 2.8, *p* <0.0001). Specific complications and quality-of-life domains were rated similarly (difference in mean 0.18, 95 % CI −0.1 to 0.4, *p* = 0.2 and difference in mean 0.2, 95 % CI −0.1 to 0.5, *p* = 0.2, respectively).

**Conclusions:**

Whilst clinicians want to measure key outcomes in trials, they rate these as less important to communicate in decision-making with patients. This discrepancy needs to be explored and addressed to maximize the impact of trials on clinical practice.

## Background

Clinical trials are a robust source of data to inform clinical decision-making. There are, however, some well described weaknesses that may undermine the interpretation of trial results. Heterogeneous measurement and reporting of outcomes, for example, can make cross-study comparisons difficult, hinder meta-analysis and lead to outcome-reporting bias. Such problems can be minimized through the establishment of a core outcome set (COS): a minimum set of outcomes that key stakeholders agree are to be measured in all trials in a particular field [1]. Many COSs have now been developed, and the benefits have been embraced internationally by research funders [1], regulatory bodies [2, 3] and journal editors [4]. Theoretically, these outcomes represent the most important aspects of an intervention and, therefore, may represent the most important issues to discuss with patients.

Communicating with patients is, however, challenging. High-quality patient-centered information is a priority for health care providers in many countries and settings [5–7], and patients generally prefer more rather than less information [8], although it is difficult to know how much is enough for an individual. One solution is to discuss very detailed information, but this may be counterproductive by overwhelming patients and reducing their understanding [9]. Patient-led communication, where discussions are guided by the individual, is helpful but patients may lack sufficient baseline knowledge to ask important questions. One method for addressing the balance between over- and under-disclosure of information is to develop a core information set (CIS) for a specific treatment. Core information represents baseline information, determined by patients and clinicians, that is necessary to stimulate further patient-centered communication [10–12]. Core information sets are now being developed in several clinical areas [11, 13].

It is hypothesized that core outcomes for trials will correspond to core information for communication in decision-making. The rationale for this is that if an outcome is considered essential to measure in a trial, then it provides information of sufficient value to use in clinical practice. Similarly, if information is necessary to communicate to patients then high-quality data supporting that information is best obtained from a well-designed and well-conducted randomized trial. This has not been previously explored. The aim of this study is to compare clinicians’ views of outcomes to include in a COS with what they consider important information for clinical practice, using colorectal cancer (CRC) surgery as an example.

## Methods

This substudy was embedded within a wider program of research to develop core outcome and information sets for CRC surgery. The scope of the COS was to agree on a minimum standard of outcomes for clinical effectiveness trials (rather than trials of treatment efficacy) of all surgical interventions for cancer of the colon and rectum. The scope of the CIS was to agree on a minimum standard of information of importance to patients and clinicians to discuss for informed consent for CRC surgery. Informed consent for recruitment to trials was not considered. Informed consent is defined by Beauchamp and Childress’ model of “autonomous authorization,” which includes the discussion of what surgeons consider to be “important” information.

The development of the core outcome and information sets was conducted according to established guidelines [1]. Phase 1: a long list of outcomes that could be measured in colorectal cancer trials, and information to communicate for consent, was identified and outcomes were categorized into domains. Phase 2: domains were operationalized into a questionnaire that was used to survey stakeholders’ views on the importance of each domain using Delphi methods. Phase 3: clinicians’ views on important outcomes for trials were compared with clinicians’ and patient’s views on important information for consent. Appropriate ethics regulatory approval was granted (National Research Ethics Service number 10/H0102/82, reviewed by South West 4 Research Ethics Committee).

- Phase 1– Domain generation
  Colorectal cancer surgical outcomes and information were identified from three sources: (1) systematic review of clinical- and patient-reported outcome literature [14, 15], (2) interviews with patients, and (3) analysis of written patient information leaflets used for colorectal surgery in UK hospitals. Duplicates were removed and a long list of outcomes/information was created. Similar outcomes/information were categorized into domains by the following process. The long list was read and re-read to facilitate immersion in these data. Categorization was conducted on an iterative item-by-item basis using the principles of constant comparison, whereby each new item was compared with those before it and placed in what was considered an appropriate category. Categories were changed, or items re-categorized appropriately, as new items were considered. After independent categorization, results were compared and discrepancies highlighted. Patient-reported outcomes were grouped into domains (e.g., ability to walk and activity levels were grouped within the physical function domain) and verified by two researchers and a patient representative [16]. Items from patient information leaflets were independently categorized by two surgeons. Discrepancies were resolved through discussion with the study lead. Overlapping domains between data sources were condensed producing a final list of domains.

**Fig. 1:**
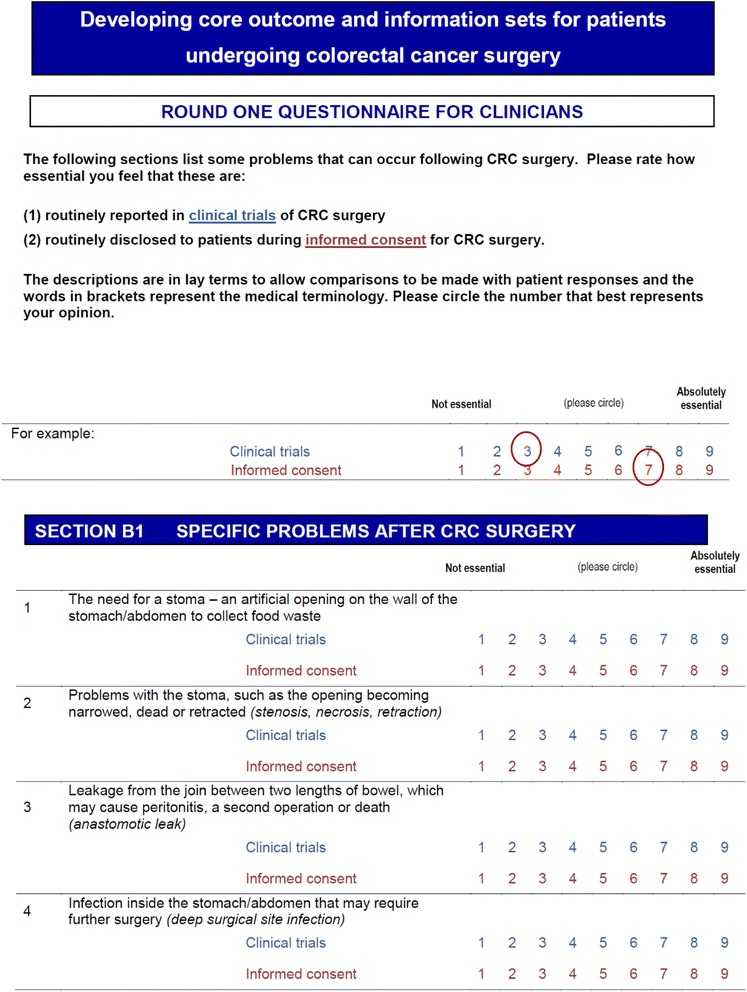
Example page from the questionnaire demonstrating the format and operationalization of the domains

The final domains were operationalized into questionnaire items using lay language with the medical terminology included in parentheses (Fig. 1). Domains were grouped into six categories: CRC-specific complications, general complications, operation and hospital stay, survival/recurrence, symptoms, and quality of life. The questionnaire was piloted by patients for face validity, understanding and acceptability and modified as a result of this feedback.

**Fig. 2:**
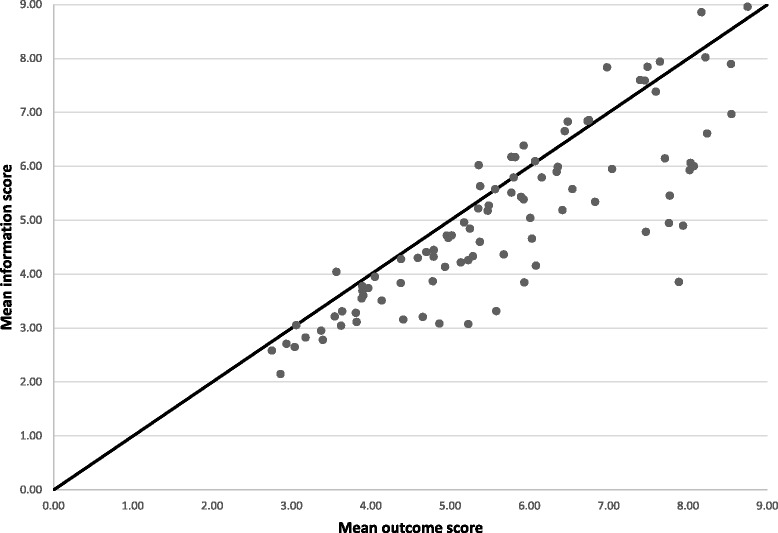
Scatter plot of mean information and mean outcome scores in round 1 with line of equality

### Phase 2 – Delphi process

The questionnaire developed in phase 1 was sent to CRC surgeons and clinical nurse specialists (round 1). Oncologists were excluded as chemo/radiotherapy was outside the scope of the COS/CIS. Surgeons and nurses were identified from UK National Health Service hospital trusts that routinely performed surgical resection of CRC and participated in the UK National Bowel Cancer Audit. Non-probabilistic purposive sampling was conducted to ensure center variation based upon geographical region (Northern England, the Midlands, South West England, South East England, and Wales), and caseload volume per annum as determined by number of major resections in 2012. Demographic details were collected including age, sex and seniority (years on the UK specialist register).

Questionnaires asked clinicians to rate the importance of (1) measuring the domains in clinical trials, and (2) discussing the domains as part of informed consent. A nine-point Likert scale was used, where 1 was “not essential,” and 9 “absolutely essential” outcome/information. Data from round 1 were collected and entered into an electronic database. Domains that were both rated between 7 and 9 (defined as “high importance”) by over 50 % of respondents *and* between 1 and 3 (defined as “low importance”) by less than 15 % were retained for round 2. The remainder were discarded. In round 2, participants were provided with feedback from round 1 in the form of their previous score for each domain and a mean score from their stakeholder group. In addition, some participants were also provided with feedback from both stakeholder groups separately (details of this exploratory substudy will be described separately). Participants were then asked to rescore each domain on the same nine-point Likert scale. Separate written informed consent was obtained from all participants.

### Phase 3 – Comparison of clinicians’ views of outcomes and information

Clinicians’ scores from rounds 1 and 2 were collated and the mean score calculated for each outcome and information domain. The difference between these means were then calculated and the domains ranked accordingly. Paired *t* tests were used to compare mean outcome and information scores of the six domain categories and as an overall score.

### Sample size

There are no agreed methods to set the sample size for Delphi surveys. Therefore, an opportunistic approach was used with the aim of obtaining approximately 100 respondents for the survey.

## Results

### Phase 1 – Domain generation

Review of all data sources identified 1216 outcomes of CRC surgery that were grouped into 100 domains. A total of six were considered only relevant to information, not trials (for example, “the risk of family members developing bowel cancer”) and were excluded from this analysis.

### Phase 2 – Delphi process

A total of 81 CRC centers were sampled, of which 63 (78 %) responded including 90 surgeons and 8 clinical nurse specialists (Table 1). The centers represented all geographical regions of England and Wales, and caseload averaged 117 major resections per year (range 38 to 275). A total of 47 domains met the criteria to be retained for round 2. The response rate in round 2 was 80 % (78/98).

**Table 1:** Participant characteristics

	Clinical centers	
	Responders (63)	Non-responders (18)
Region (%)		
Northern England	14 (22)	5 (28)
Midlands	8 (13)	0
South East England	22 (35)	10 (55)
South West England	9 (14)	0
Wales	10 (16)	3 (17)
Mean number of major colorectal resections (range)^a^	117 (38 to 275)	90 (29 to 210)
	Participants	
	Responders (94)	
Male sex (%)	70 (75)	
Post held (%)		
Surgeon	86 (91)	
Nurse	8 (9)	
Time in post (%)		
<5 years	12 (14)	
5 to 10 years	24 (28)	
>10 years	49 (58)	
Age		
31–40	8 (9)	
41–50	48 (51)	
51–60	34 (36)	
Over 60	4 (4)	

^a^Number of major cancer resections are defined by the UK National Bowel Cancer Audit 2012

### Phase 3 – Comparison of clinicians’ views of outcomes and information

In the first survey, clinicians considered most domains (76/94; 81 %) of greater importance to measure as trial outcomes than to communicate to patients (Fig. 2). The greatest difference was with the number of lymph nodes harvested during the operation (difference in mean 4.1, 95 % CI 3.5 to 4.7), unplanned re-admission to hospital (difference in mean 3.1, 95 % CI 2.5 to 3.6) and survival (difference in mean 2.8, 95 % CI 2.2 to 3.4). Of the 18 domains that were considered more important to communicate to patients than to measure in trials, rates of superficial surgical site infection (difference in mean −0.9, 95 % CI −1.1 to −0.5) and the need for a stoma (difference in mean −0.7, 95 % CI −0.9 to −0.4) showed the greatest difference. Paired *t* tests demonstrated that generic complications, the operation and hospital stay, survival/recurrence and symptom domain categories were all scored significantly higher as outcomes in trials rather than information for clinical practice (Table 4). The overall effect was that clinicians rated outcomes higher than information (difference in mean 0.7, 95 % CI 0.4 to 0.9, *p* <0.0001).

**Table 2:** The 20 domains with the greatest difference between mean outcome and information scores in round 1

Domain name	Category	Mean outcome score	Mean information score	Difference in mean	95 % CI
Lymph node harvest	Operation and hospital stay	7.89	3.85	4.07	3.46 to 4.68
Unplanned re-admission	Operation and hospital stay	7.94	4.90	3.06	2.52 to 3.61
Long-term survival	Survival/recurrence	7.76	4.95	2.81	2.21 to 3.41
Disease-free interval	Survival/recurrence	7.47	4.78	2.72	2.13 to 3.30
Length of hospital stay	Operation and hospital stay	7.77	5.45	2.35	1.83 to 2.88
Second primary cancer	Survival/recurrence	5.58	3.31	2.27	1.67 to 2.88
Length of bowel removed	Operation and hospital stay	5.23	3.07	2.16	1.57 to 2.75
Distant recurrence	Survival/recurrence	8.02	5.93	2.11	1.57 to 2.64
Operative time	Operation and hospital stay	5.94	3.84	2.07	1.54 to 2.61
Local recurrence	Survival/recurrence	8.07	6.00	2.04	1.54 to 2.55
Recurrence	Survival/recurrence	8.03	6.06	1.99	1.49 to 2.49
Length of time after surgery until the bowels open	Operation and hospital stay	6.08	4.15	1.98	1.46 to 2.50
Equipment failure	Operation and hospital stay	4.86	3.08	1.79	1.21 to 2.37
Reoperation	Operation and hospital stay	8.24	6.61	1.64	1.16 to 2.11
Resection margins	Operation and hospital stay	8.55	6.97	1.60	1.06 to 2.14
Non-progression	Operation and hospital stay	7.71	6.15	1.56	1.06 to 2.07
Multi-organ failure	Generic complications	6.83	5.34	1.55	1.03 to 2.07
Allergic reactions	Generic complications	4.66	3.21	1.45	0.87 to 2.03
Enterocutaneous fistula	CRC-specific complications	6.03	4.66	1.39	0.88 to 1.90
Enterovisceral fistula	CRC-specific complications	5.68	4.36	1.27	0.78 to 1.76

Results of the second survey were moderated compared to round 1. Only 24/47 (51 %) outcome domains were rated higher than the corresponding information domains. The greatest difference was still evident in the number of lymph nodes harvested during the operation (difference in mean 3.4, 95 % CI 2.8 to 4.0), survival (difference in mean 2.5, 95 % CI 1.9 to 3.0) and unplanned re-admission to hospital (difference in mean 2.4, 95 % CI 1.9 to 3.0). Paired *t* tests demonstrated that outcomes regarding the operation and hospital stay, and survival/recurrence remained more important than information on the same topics. In this round, clinicians thought CRC-specific complications were more important as information to discuss with patients then outcomes of trials, but the difference was small (difference in mean −0.3, 95 % CI −0.5 to −0.1, *p* <0001). The overall effect favored outcomes rather than information (Table 4).

**Table 3:** The 20 domains with the greatest difference between mean outcome and information scores in round 2

Domain name	Category	Mean outcome score	Mean information score	Difference in mean	95 % CI
Lymph node harvest	Operation and hospital stay	7.79	4.40	3.36	2.75 to 3.97
Long-term survival	Survival/recurrence	7.91	5.42	2.45	1.94 to 2.96
Unplanned re-admission	Operation and hospital stay	7.82	5.37	2.41	1.87 to 2.96
Disease-free interval	Survival/recurrence	7.57	5.13	2.39	1.84 to 2.95
Local recurrence	Survival/recurrence	8.04	6.13	1.90	1.40 to 2.40
Recurrence	Survival/recurrence	8.08	6.23	1.86	1.34 to 2.38
Length of hospital stay	Operation and hospital stay	7.51	5.63	1.86	1.33 to 2.38
Distant recurrence	Survival/recurrence	8.03	6.31	1.70	1.18 to 2.23
Length of bowel removed	Operation and hospital stay	4.88	3.29	1.60	1.00 to 2.20
Length of time after surgery until the bowels open	Operation and hospital stay	5.77	4.36	1.43	0.93 to 1.93
Non-progression	Operation and hospital stay	8.03	6.57	1.43	0.93 to 1.93
Resection margins	Operation and hospital stay	8.70	7.36	1.33	0.91 to 1.75
Reoperation	Operation and hospital stay	8.08	7.01	1.04	0.63 to 1.46
Length of time after surgery to start eating and drinking	Operation and hospital stay	6.25	5.47	0.80	0.33 to 1.27
Overall quality of life	Quality of life	6.61	5.97	0.66	0.30 to 1.02
Operative blood loss	Generic complications	6.32	5.73	0.61	0.12 to 1.09
Operative mortality	Operation and hospital stay	8.69	8.11	0.57	0.20 to 0.95
Overall health	Quality of life	5.78	5.37	0.43	0.02 to 0.84
General pain	Symptoms	6.04	5.89	0.17	−0.22 to 0.55
Conversion to open operation	Operation and hospital stay	7.67	7.61	0.07	−0.16 to 0.31

**Table 4:** Comparison of mean outcome and information scores for domain categories

Round 1							
Domain category	Number of domains	Mean outcome score	Mean information score	Difference in means	SD	95 % CI	*P* value^a^
CRC-specific complications	14	6.17	5.99	0.18	1.29	−0.08 to 0.44	0.18
Generic complications	17	5.41	4.82	0.59	1.70	0.25 to 0.93	0.001
Operation and hospital stay	15	7.09	5.39	1.70	1.50	1.40 to 2.00	<0.0001
Survival/recurrence	6	7.49	5.15	2.34	2.28	1.88 to 2.80	<0.0001
Symptoms	29	4.55	4.27	0.29	1.29	0.03 to 0.55	0.03
Quality of life	13	5.26	5.06	0.20	1.68	−0.14 to 0.54	0.24
Overall	94	5.65	4.99	0.66	1.12	0.43 to 0.88	<0.0001
Round 2							
CRC-specific complications	6	7.44	7.74	−0.30	0.77	−0.48 to −0.12	<0.0001
Generic complications	5	6.48	6.66	−0.17	1.48	−0.52 to 0.18	0.32
Operation and hospital stay	12	7.40	6.07	1.33	1.20	1.04 to 1.62	<0.0001
Survival/recurrence	5	7.90	5.84	2.06	1.98	1.59 to 2.53	<0.0001
Symptoms	10	5.76	6.00	−0.24	1.16	−0.52 to 0.03	0.08
Quality of life	9	5.62	5.66	−0.04	1.28	−0.34 to 0.26	0.80
Overall	47	6.66	6.23	0.44	0.87	0.23 to 0.64	0.0001

^a^Paired *t* test

## Discussion

This study demonstrated that clinicians consistently rated several health outcome domains as more important to measure in trials than to use as information provision for clinical decision-making with patients. The effect was most pronounced in the first survey round (which does not show views of others) where significant differences were observed regarding outcomes relating to the operation and hospital stay, and survival/recurrence. In the second survey (where views of patients and other health professionals are presented for the participant to re-score the domains) the effect was similar although attenuated. It is possible that health professionals’ really value trial outcomes differently to those of importance for use for decision-making. In which case, it is important to consider whether trial outcomes should be more representative of information that is valued. Findings also suggest that clinicians’ value outcomes that they find difficult to discuss with patients, in which case training in communication and shared decision-making is required. These discrepancies are important to explore further to maximize the impact of trials on clinical practice.

It has not been possible to identify previous studies that have directly compared clinician’s views on outcomes for trials and views on information for clinical practice. A recent study, however, has developed a CIS for oesophageal cancer surgery [11]. Detailed comparisons with this work were not possible because only limited data are presented in the publication; however, clinicians’ views appear to be similar to those found here. Operation-specific complications, such as anastomotic leak, and peri-operative mortality were rated highly, and long-term survival information did not feature in the top 20 issues of importance to clinicians. The reasons for these findings are not clear. Multiple previous studies have shown that cancer patients have detailed and broad information needs, especially regarding issues of prognosis [8]. A survey of 2300 cancer patients in the UK, for example, demonstrated that over 95 % wanted to know survival statistics [17]. Clinicians’ views appear discordant with this evidence. It may be that clinicians consider the information upsetting for patients or difficult to understand. Perhaps clinicians value the data generated from randomized trials to make treatment recommendations, but do not consider it important to discuss with patients.

It has been widely recognized that patients and professionals differ with regards to what outcomes are considered important to measure in clinical trials. Core outcome set development was first pioneered in the field of rheumatology, where the OMERACT (Outcome Measures in Rheumatology) initiative started using consensus methodology to enable professionals to agree on which outcomes to measure in trials. This was initially a closed process, but it was opened up to patients after 6 years. Patients identified three outcome domains that were not considered important by professionals (fatigue, sleep disturbance and arthritic “flairs”) and these were subsequently included in the core set. Although not explicit, it is likely that patients were interested in discussing these trial outcomes in clinical consultations. This is, however, the first study to directly measure the internal discrepancies between what clinicians want to measure in trials and what they want to communicate to patients.

Methods used in this study followed guidelines established by the Core Outcome Measures in Effectiveness Trials (COMET) initiative to develop a COS, but there are some weaknesses. The validity of including specific outcome/information domains in the questionnaires can be criticized; however, this has no impact on the comparative importance that clinicians and patients place on such outcomes/information. Rather, it represents a limitation of the development of the COS. Another limitation is that the findings may represent a failure in methodology and, in particular, the way the questionnaire was presented may have biased responses. For example, participants were asked to rate domains as outcomes before rating them as information for decision-making. This may have induced the prioritization of the former over the latter. Similarly, the order of the domains within the questionnaire may have had an impact on responses. Domains regarding quality of life came at the end of the questionnaire, and thus may have been seen as of lower priority. This, however, should not have influenced the comparative scores of individual domains. Descriptive statistics were used to summarize domain scoring, and multiple hypothesis testing was avoided. Caution should be exercised, however, when comparing individual domain scores because of issues of multiplicity.

Further research is needed to understand the implications of this study. Understanding why clinicians rate these outcome and information domains differently is critical. Qualitative interviews with clinicians and triallists would provide a framework for exploring these issues. It is also important to understand the impact of integrating patients’ views of outcome and information into core set development. It may be that patients’ views mitigate some of the discrepancies evident in this study, but others may become apparent. Research is then needed to identify methods to harmonize the measurement of outcomes in trials and their use in clinical practice.

## Conclusion

This study demonstrates that whilst clinicians want to measure key outcomes in trials, they rate these as less important communication in decision-making with patients. This discrepancy needs to be explored and addressed to maximize the impact of trials on clinical practice.

## Abbreviations

CI, confidence interval; CIS, core information set; COS, core outcome set; CRC, colorectal cancer
